# Serum cytokine dysregulation signatures associated with COVID-19 outcomes in high mortality intensive care unit cohorts across pandemic waves and variants

**DOI:** 10.1038/s41598-024-64384-y

**Published:** 2024-06-13

**Authors:** Henrike Maaß, Mario Ynga-Durand, Marko Milošević, Fran Krstanović, Marina Pribanić Matešić, Iva Žuža, Stipan Jonjić, Ilija Brizić, Alan Šustić, Frank Bloos, SepNet Critical Care Trials Group, Gernot Marx, Ulrich Jaschinski, Konrad Reinhart, Claudia Spies, Lorenz Reil, Christian Putensen, Maximilian Ragaller, Stefan Utzlino, Onnen Mörer, Matthias Gründling, Stefan Kluge, Axel Nierhaus, Tobias Welte, Michael Bauer, Frank Bloos, Katrin Ludwig, Michael Kiehntopf, Gunnar Elke, Holger Bogatsch, Christoph Engel, Markus Loeffler, Josef Briegel, Ines Kaufmann, Stefan John, Reimer Riessen, Patrick Meybohm, Alen Protić, Luka Čičin-Šain

**Affiliations:** 1 Department of Viral Immunology, Helmholtz Center for Infection Research, Braunschweig, Germany; 2 Centre for Individualized Infection Medicine (CiiM), a joint venture of Helmholtz Centre for Infection Research and Hannover Medical School, Hannover, Germany; 3 Department of Anesthesiology, Faculty of Medicine, Reanimation, Intensive Care and Emergency Medicine, University of Rijeka, Rijeka, Croatia; 4 Faculty of Medicine, Center for Proteomics, University of Rijeka, Rijeka, Croatia; 5 Department of Radiology, Clinical Hospital Centre Rijeka, Rijeka, Croatia; 6 Department of Clinical Medical Science II, Faculty of Health Studies, University of Rijeka, Rijeka, Croatia; 7 Department of Anesthesiology and Intensive Care Medicine, Jena University Hospital, Jena, Germany; 8 German Centre for Infection Research (DZIF), Partner Site Hannover/Braunschweig, Braunschweig, Germany; 9 Department of Surgical Intensive Care Medicine and Intermediate Care, University Hospital RWTH Aachen, Aachen, Germany; 10 Department of Anesthesiology and Surgical Intensive Care Medicine, University Hospital Augsburg, Augsburg, Germany; 11 Division of Operative Intensive Care Medicine, Department of Anesthesiology, Charité Berlin, Berlin, Germany; 12 Department of Cardiology and Intensive Care Medicine, Vivantes Hospital Neukölln, Berlin, Germany; 13 Department of Anesthesiology and Intensive Care Medicine, Division of Intensive Care Medicine, University Hospital Bonn, Bonn, Germany; 14 Department of Anesthesiology and Intensive Care Medicine, University Hospital Carl Gustav Carus, Dresden, Germany; 15 Department of General Surgery, University Hospital Freiburg, Freiburg, Germany; 16 Department of Anesthesiology, University Medical Center, Georg-August-University Göttingen, Göttingen, Germany; 17 Depatment of Anesthesiology and Intensive Care Medicine, University Hospital Greifswald, Greifswald, Germany; 18 Department of Intensive Care Medicine, University Hospital Hamburg-Eppendorf, Hamburg, Germany; 19 Department of Pneumology and Infectiology, University Hospital Hannover, Hannover, Germany; 20 Department of Anesthesiology and Intensive Care Medicine, Jena University Hospital, Jena, Germany; 21 Institute of Clinical Chemistry and Laboratory Diagnostics, Jena University Hospital, Jena, Germany; 22 Department of Anesthesiology and Intensive Care Medicine, University Hospital Schleswig-Holstein – Campus Kiel, Kiel, Germany; 23 Center for Clinical Studies, University Lepizig, Leipzig, Germany; 24 Department of Anesthesiology, University Hospital Munich, Munich, Germany; 25 Department of Internal Medicine, Division Cardiology, Hospital Nuremberg, Nuremberg, Germany; 26 Department of Internal Medicine, University Hospital Tübingen, Tübingen, Germany; 27 Department of Anaesthesiology, Intensive Care, Emergency and Pain Medicine, University Hospital Würzburg, Würzburg, Germany

**Keywords:** COVID-19, SARS-CoV-2, Cytokines, Biomarker, Variant of concern, Mortality, SARS-CoV-2, Infectious diseases, Biomarkers, Outcomes research

## Abstract

The aim of this study was to characterize the systemic cytokine signature of critically ill COVID-19 patients in a high mortality setting aiming to identify biomarkers of severity, and to explore their associations with viral loads and clinical characteristics. We studied two COVID-19 critically ill patient cohorts from a referral centre located in Central Europe. The cohorts were recruited during the pre-alpha/alpha (November 2020 to April 2021) and delta (end of 2021) period respectively. We determined both the serum and bronchoalveolar SARS-CoV-2 viral load and identified the variant of concern (VoC) involved. Using a cytokine multiplex assay, we quantified systemic cytokine concentrations and analyzed their relationship with clinical findings, routine laboratory workup and pulmonary function data obtained during the ICU stay. Patients who did not survive had a significantly higher systemic and pulmonary viral load. Patients infected with the pre-alpha VoC showed a significantly lower viral load in comparison to those infected with the alpha- and delta-variants. Levels of systemic CTACK, M-CSF and IL-18 were significantly higher in non-survivors in comparison to survivors. CTACK correlated directly with APACHE II scores. We observed differences in lung compliance and the association between cytokine levels and pulmonary function, dependent on the VoC identified. An intra-cytokine analysis revealed a loss of correlation in the non-survival group in comparison to survivors in both cohorts. Critically ill COVID-19 patients exhibited a distinct systemic cytokine profile based on their survival outcomes. CTACK, M-CSF and IL-18 were identified as mortality-associated analytes independently of the VoC involved. The Intra-cytokine correlation analysis suggested the potential role of a dysregulated systemic network of inflammatory mediators in severe COVID-19 mortality.

## Introduction

COVID-19 is an infectious disease caused by the severe acute respiratory syndrome virus 2 (SARS-CoV-2). Although respiratory failure is the most prominent feature associated with severe COVID-19^1^, it has been demonstrated that systemic hyperinflammation also contributes to negative outcomes, and a distinct immune profile has been reported^2–4^. Several cytokines associated with unfavorable prognosis have been detected^5–10^, and specific anti-cytokine therapies have proven beneficial in severe COVID-19^11,12^. Despite these advances, cytokine identification as biomarkers has not been consistent across studies, possibly due to differences in population characteristics, detection assays and the time of sampling^13^. This last factor may be critical as most of the reports analyze samples retrieved at the time of hospital admission^6,8,9,14,15^, and they do not offer a direct insight into the immune activation landscape of critically ill patients. Moreover, hospital admission criteria are not uniform and depend on healthcare availability and local guidelines. Importantly, the majority of studies focused on relatively healthy cohorts that include non-critically ill patients with low mortality rates and have relied on clinical evaluation to classify for severity and outcomes. In order to develop adequate prognostic tools and/or target candidates, well-defined homogenous cohorts with clear classification endpoints are important.

For this report, we had access to critically ill COVID-19 patient cohorts with a high mortality outcome at two different stages during the pandemic, from a third-level referral center for COVID-19 patients in Croatia. Patient recruitment for the first cohort took place during the period when pre-alpha and alpha SARS-CoV-2 variants of concern (VoC) were dominant and will be referred to as the pre-delta cohort. The second cohort was recruited during the SARS-CoV-2 delta VoC wave in late 2021 and will subsequently be referred to as the delta cohort. From both cohorts, we analyzed serum and bronchoalveolar samples collected at admission to the intensive care unit (ICU) and during their hospitalization. This population was mainly composed of severely ill and/or rapidly deteriorating patients undergoing invasive mechanical ventilation (IMV) with a 30-day post-admission mortality of 70.9% and 72.7% for the pre-delta and the delta cohort respectively. We analyzed the systemic cytokine levels across time, detecting a systemic immune signature associated with higher disease severity and a fatal outcome. Associations with SARS-CoV-2 viral load and clinical characteristics were also investigated, suggesting an early dysregulated immune profile present at ICU admission in non-survivors compared to survivors.

## Material and methods

### Study participants

The study population included two cohorts. The first cohort (pre-delta cohort) included 54 patients admitted to the COVID-19 Intensive Care Unit (ICU) of the Clinical Hospital Center Rijeka with the diagnosis of acute respiratory distress syndrome (ARDS) defined by the Berlin criteria^16^ and severe COVID-19 (established by clinical assessment^17^ and initial viral detection by nasopharyngeal testing) from November 2020 to April 2021. These patients required invasive ventilator support, and ICU specialists followed standardized treatment guidelines. A non-ICU group of eighteen patients (hospitalized oxygen-dependent SARS-CoV-2 positive patients who did not require invasive respiratory support) was included as a control. A second cohort of 33 patients (delta cohort) was used to validate the findings obtained from the first cohort. Patients from the delta cohort were admitted to the ICU between November and December of 2021. The treatment guidelines were maintained across the two cohorts, in which all patients received steroid treatment (methylprednisolone or dexamethasone) in accordance with international guidelines. A more detailed description of the patient cohort was provided in our previous publication^18^. No power analysis was done due to a lack of reported effect size under matching or similar conditions of high mortality. Sample size was based on patient availability and sample collection was maintained during COVID-19 pandemic waves.

To provide a comparison to critically-ill patients without SARS-CoV-2 infection, serum samples from patients with non-COVID pneumonia and severe sepsis or septic shock were obtained from the randomized controlled trials SISPCT and MAXSEP^19,20^ of the SepNet Critical Care Trials Group. Patients were selected based on age, comorbidities, and stage of critical illness, to be comparable to COVID-19 cohorts. Serum samples obtained on the day of the start of invasive ventilator support were analyzed and patients were classified according to their 28-day post-admission mortality.

### Clinical data and outcomes

Clinical characteristics of the patients from the pre-delta cohort are listed in and in a previous report Ynga-Durand et al*.*^18^. Characteristics of the delta cohort and the non-COVID cohort are listed in Table S1 and Table S2, respectively.

Clinical data were obtained from electronic medical records. Rijeka Hospital Medical constituted a highly specialized referral centre for severe COVID-19 cases in Primorsko-goranska, Licko-senjska and Istarska counties in Croatia, and close follow-up of individuals using the hospital information system (IBIS) or via phone calls was maintained up to 60 days. No variables with missing data were included in this study, except for days since symptom onset and lung imaging data.

### Samples collection

#### Serum samples

Serum samples were obtained within the first 36 h after intubation and every two to three days until the 10th day of ICU stay (unless earlier ICU discharge or death occurred). Whole blood samples were transported to the Center for Proteomics of the University of Rijeka for initial processing. Samples were incubated for 30–60 min at room temperature until a blood clot was visible. Serum and blood clot were separated and the serum was centrifuged at 1500 g for 10 min. The supernatant was aliquoted and stored at − 20 °C. The samples were shipped on dry ice to the Helmholtz Center for Infection Research in Braunschweig and stored at −80 °C until analysis.

#### Bronchoalveolar lavage (BAL) samples

BAL samples were taken at the same time points as the serum samples. 10 mL of sterile saline was instilled in the main right bronchus through the endotracheal tube (ET). The fluid was aspirated until at least 5 mL of aspirate was collected. Afterwards, the samples were initially processed in the Center for Proteomics of the University of Rijeka by filtration through a 100 µm cell strainer and centrifugation at 400 g at 4 °C for 7 min to remove debris and mucous strands. The supernatant was aliquoted and stored at – 80 °C and shipped to the Helmholtz Center for Infection Research in Braunschweig on dry ice.

### Quantification of serum cytokines

Serum samples were pre-processed by centrifugation at 800 g and 4 °C for 7 min. Serum proteins were measured using the Bio-Plex Pro™ Human Cytokine Screening Panel, 48-Plex (Bio-Rad, Hercules, USA, 10,000,092,045) following manufacturer’s instructions. The assay was performed using the Bio-Plex 200 system and analyzed with the Bio-Plex Manager™ version 6.2 software (Bio-Rad, Hercules, USA). Measurements below detection limit were set to the lowest standard value, values above detection limit were set to the highest standard concentration. Cytokines that showed levels below detection limit in more than 50% of measured samples were not included in the statistical analysis.

### Isolation and quantification of viral RNA from serum and BAL samples

RNA was isolated and quantified as previously described^18^. In brief, RNA was isolated from 200 µl of serum or BAL using the Innuprep Virus DNA/RNA virus kit (Analytic Jena, Germany 845-KS-4710250). SARS-CoV-2 nucleocapsid protein N2 and the host housekeeping gene RNase P were quantified by reverse-transcriptase quantitative PCR (RT-qPCR) using the TaqPath™ 1-Step RT-qPCR Master Mix (ThermoFisher, Cat A15300). 5 µl of isolated RNA and serially diluted SARS-CoV-2 plasmid controls were used for RNA quantification (Integrated DNA Technologies, Cat # 10,006,625). Viral copy number calculation was performed by OneStep qPCR Software (Thermo Fisher Scientific, Waltham, MA, USA).

SARS-CoV-2 variants were identified using the GSD NovaType II (for pre-delta cohort) and IV (for delta cohort) SARS-CoV-2 RT-PCR assays (PCOV6083T, PCOV6191T, Gold Standard Diagnostics Europe, Dietzenbach, Germany). These assays allow the simultaneous measurement of the mutations K417N, E484K and N501Y or K417N, E484K and L452R in the spike gene. The assay was performed by following the manufacturer’s instructions and using the Light Cycler® 480 (Roche, Basel, Switzerland). Two patients from the pre-delta and one patient from the delta cohort were considered inconclusive due to low viral load.

### Statistical analysis

The Shapiro–Wilk test was used to determine normality. Kruskal–Wallis test with Dunn’s correction or unpaired Mann–Whitney test was used for group comparisons where appropriate. Spearman’s rank or Pearson correlation test was used for correlation analysis where appropriate. Data representation was performed using R software v 4.1.2 using the packages heatmaply^21^, corrplot^22^, complexHeatmap^23^, pheatmap^24^ and upsetR^25^, and GraphPad Prism v 9.3.0 (GraphPad Software, San Diego, California USA).

### Ethics approval

The study protocol was approved by the Institutional Review Board of the Rijeka Clinical Hospital Center (2170-29-02/1-20-2). Written informed consent was waived by the Ethics Committee of the Rijeka Clinical Hospital Center, as all procedures associated to sample collection were part of ICU routine care according to institutional guidelines and the study was performed in a retrospective manner.

## Results

### Demographic and clinical characteristics of the study population

For the pre-delta cohort, fifty-four patients with severe COVID-19 and ARDS requiring invasive mechanical ventilation, and eighteen hospitalized non-ICU patients requiring supplemental oxygen (but no mechanical ventilation) were included. A summary of the clinical characteristics of the ICU (classified by 30-day post admission survival outcome) and non-ICU population is shown (Table 1). The ICU non-survivor group was significantly older than the ICU survivor group and had a higher ICU severity score at admission (SOFA and APACHE II). Additionally, all non-survivors and 75% of survivors presented with moderate to severe ARDS at their admission to ICU. No significant differences were observed in the occurrence of comorbidities or days on invasive mechanical ventilation.

**Table 1:** Demographic and clinical characteristics of the patients included in this study, classified by their ICU stay outcome ^18^.

	ICU non-survival (n = 38)	ICU survival (n = 16)	Non-ICU symptomatic (n = 16)	ρ between non-survival and survival groups
Demographic characteristics				
Female %	31.58	25	25	ns^a^
Age in years (Mean ± SD)	71 ± 9.87	64 ± 10.15	70 ± 14.25	0.023^b^
Sample per patient	1.87 ± 0.93	1.94 ± 0.93	1	ns^c^
Clinical characteristics				
PaO_2_/FiO_2_ at ICU admission (Median, p25-p75)	93 (83.5–109.8)	159.5 (128–191.8)	–	< 0.0001^c^
SOFA score at admission (Median, p25-p75)	7 (6–9)	4.5 (3.25–5)	–	< 0.0001^c^
APACHE II score at admission (Median, p25-p75)	16 (15–22)	10.5 (8.25–13)	–	< 0.0001^c^
SARS-CoV-2 Immunization started (%)	0	0	6.25	ns^a^
Comorbidities				
Coronary heart disease (%)	23.68	12.5	18.75	ns^a^
Hypertension (%)	76.32	68.75	62.5	ns^a^
Diabetes (%)	39.47	18.75	25	ns^a^
Obesity (%)	26.32	43.75	12.5	ns^a^
Cancer (%)	5.26	12.5	12.5	ns^a^
Chronic respiratory disease (%)	15.79	18.75	6.25	ns^a^
Immunosuppression (%)	2.63	0	0	ns^a^
Number of comorbidities (Median, p25-p75)	2 (1–3)	2 (1–2.75)	1 (0–2.75)	ns^c^

ns = non-significant.

^a^Calculated by Fisher’s exact test.

^b^Calculated by unpaired t test with Welch’s correction.

^c^Calculated by Mann–Whitney test.

Detailed demographic data of the delta cohort is listed in Table S1. A comparison of the pre-delta cohort to the delta cohort showed similar characteristics, except for the days under mechanical ventilator support (Table 2). Importantly, none of the pre-delta and only three of the delta (one survivor, two non-survivors) patients had started their vaccinations against SARS-CoV-2. Due to the small number of cases, we have not examined vaccination effects.

**Table 2:** Comparison of demographic and clinical data between pre-delta cohort and delta cohort (main vs validation cohort).

	Pre-delta cohort	Delta cohort	ρ
	(n = 54)	(n = 33)	
Survival %	29.63	27.27	ns^a^
Female %	29.63	33.33	ns^a^
Age in years (mean)	67.8	64.21	ns^b^
PaO_2_/FiO_2_ at ICU admission (Median, p25–p75)	102.5 (86.75–140.3)	126 (100–158.5)	ns^c^
SOFA score at admission (Mean ± SD)	6.65 ± 2.22	6.24 ± 2.36	ns^b^
APACHE II score at admission (Median, p25–p75)	15 (12.75–18.25)	17 (11.5–21.5)	ns^c^
SARS-CoV-2 Immunization started (%)	0	9.09	ns^a^
Number of comorbidities (Median, p25-p75)	2 (1–3)	2 (1–2)	ns^c^
CRP mg/dL (Median, p25–p75)	144.7 (66.43–192.7)	163.8 (102.4–235.6)	ns^c^
Days since symptom onset until hospital admission (Median, p25-p75)	9 (7.5–11.5)	7 (6–11)	ns^c^
Days on mechanical ventilation (Median, p25–p75)	10 (5.75–14)	6 (4–9)	0.0295^c^

ns = non-significant.

^a^Calculated by Fisher’s exact test.

^b^Calculated by unpaired t test with Welch’s correction.

^c^Calculated by Mann–Whitney test.

### Comparison of viral load of pre-alpha, alpha and delta VoC infected patients

We confirmed the VoC identity for the pre-delta cohort and delta cohort samples by variant analysis except for one sample. We measured serum and BAL viral loads of both cohorts at ICU admission (earliest sample available) and during ICU stay. We found that patients from the delta cohort who did not survive had a higher serum and BAL viral load at ICU admission (“earliest”) (Fig. S1a), similar to our previous dataset limited to the pre-delta cohort^18^ after age-adjustment. We compared BAL and serum viral loads from the earliest sample harvested after ICU admission, and the highest load measured during ICU stay to identify if viral replication was different among detected VoCs. The viral load in pre-alpha VoC infected patients was significantly lower than in alpha infected patients, except for earliest serum measurements. The viral load in delta patients did not significantly differ from either, except in the highest measured burden in BAL which was significantly higher than pre-alpha VoC (Fig. S1b).

### Serum cytokine concentration at ICU admission reveals a spectrum of systemic responses in severe COVID-19

**Figure 1:**
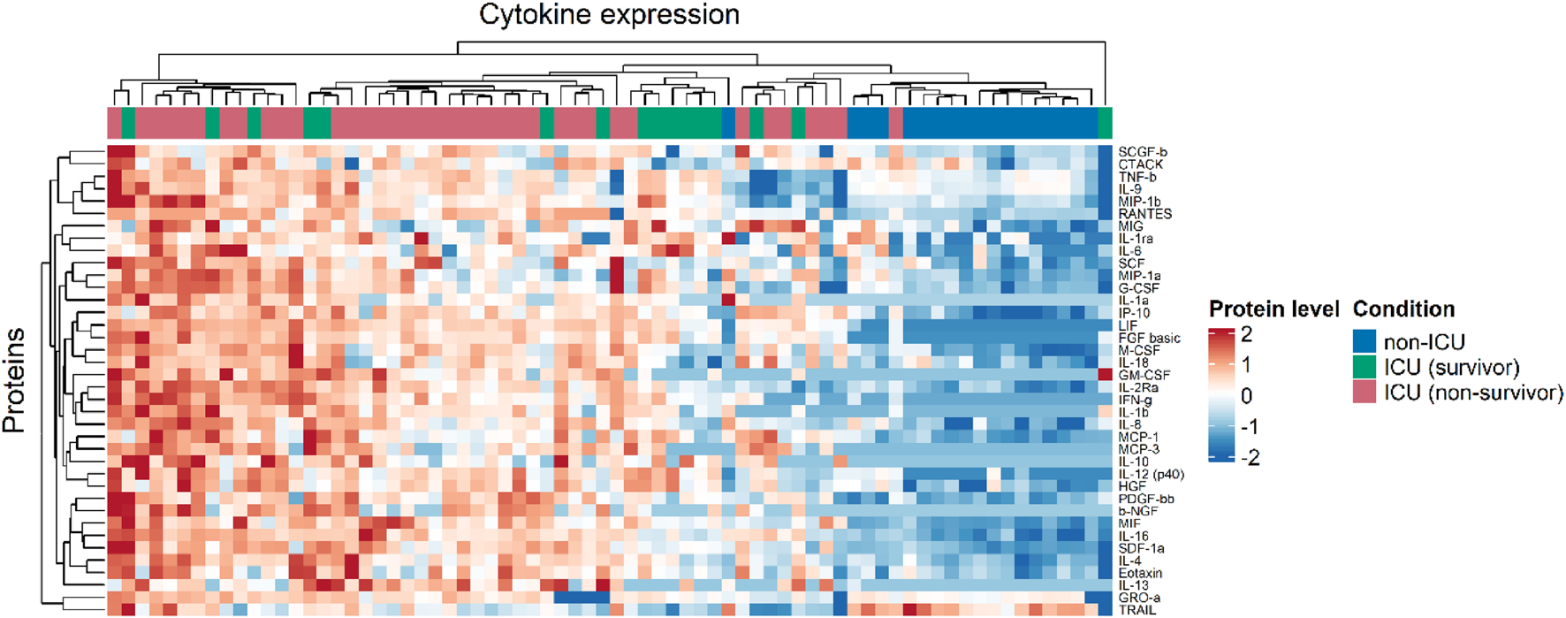
Cytokine analysis of hospitalized COVID-19 patients. Heatmap of log-transformed cytokine concentrations of samples of the earliest time points. Patients are represented per column and patient conditions are indicated by column annotations. Cytokines are represented by rows. Clustering was performed based on Euclidean distance.

To investigate the systemic immune response against SARS-CoV-2 at the time of critical deterioration, we studied serum samples collected at ICU admission and measured 48 cytokine, chemokine and growth factor biomarkers. Based on the 30-day post-admission mortality, the ICU group was divided into survivors and non-survivors. Unbiased patient clustering based on analyte quantification of the pre-delta cohort revealed a spectrum of systemic responses, where the majority of non-ICU patients grouped together showing lower serum concentrations (except for TRAIL) in comparison to ICU survivors and non-survivors (Fig. 1). A similar clustering pattern was observed in the delta cohort (Fig. S2) in which the majority of patients who did not survive had higher analyte levels in comparison to surviving patients.

### A subset of cytokines are associated to increased systemic inflammation, clinical severity, serum viral loads and mortality

**Figure 2:**
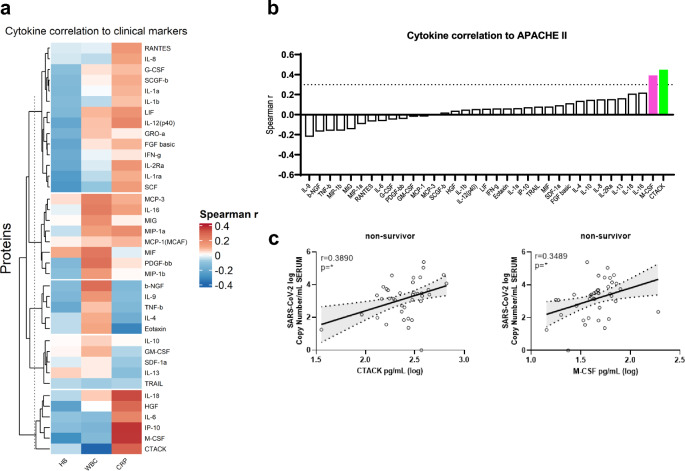
Cytokine association to clinical marker. (**a**) Spearman correlation analysis of cytokines to hemoglobin (HB) values, white blood cell count (WBC) and C reactive protein (CRP) was performed. Red indicates positive correlation, blue negative correlation. (**b**) Waterfall plot of Spearman correlation from cytokines to APACHE II scores. The dotted line indicates values above r_S_ = 0.3 and cytokines with statistically significant correlation (M-CSF (magenta) and CTACK (green)). (**c**) Correlation of CTACK and M-CSF to SARS-CoV-2 viral load in serum samples of ICU non-survivors (black). Pearson analysis was performed. Linear-regression is shown as well as the 90% CI indicated by the dotted lines. ns = non-significant; * = *p* < 0.05.

In order to analyze the implications of elevated systemic cytokine levels, we investigated the association of serum analytes concentrations to clinical characteristics at ICU admission. Remarkably, CTACK, M-CSF, IP-10, IL-6, HGF and IL-18 showed a strong positive correlation to C Reactive Protein (CRP), a systemic inflammation biomarker, in comparison to other cytokines (Fig. 2a). Additionally, M-CSF (r_S_ = 0.3918, *p* = 0.0034) and CTACK (r_S_ = 0.4482, *p* = 0.0007) showed positive association with the APACHE II score, a clinically validated severity score and mortality estimation tool^26^ (Fig. 2b).

As our previous work has demonstrated that non-survivor patients have higher systemic SARS-CoV-2 loads^18^, we investigated if cytokine concentrations were associated with higher viral copy numbers. Surprisingly, both M- CSF and CTACK showed a weak but significant correlation to serum SARS-CoV-2 copy numbers at ICU admission in the group of ICU non-survivors (Fig. 2c). The same analysis of the delta cohort revealed a distinct pattern of cytokine correlation to WBC, RBC or CRP (Fig. S3, a) in comparison to the pre-delta cohort. Notably, despite these differences, the positive correlation of CTACK to APACHE II (r_S_ = 0.51938, *p* = 0.002) (Fig. S3b) and to SARS-CoV-2 viral load in non-survivors (Fig. S3, c) was maintained. This indicates differences in cytokine responses between patients infected with pre-delta and delta VoCs, but also consistent associations across variants analysis.

**Figure 3:**
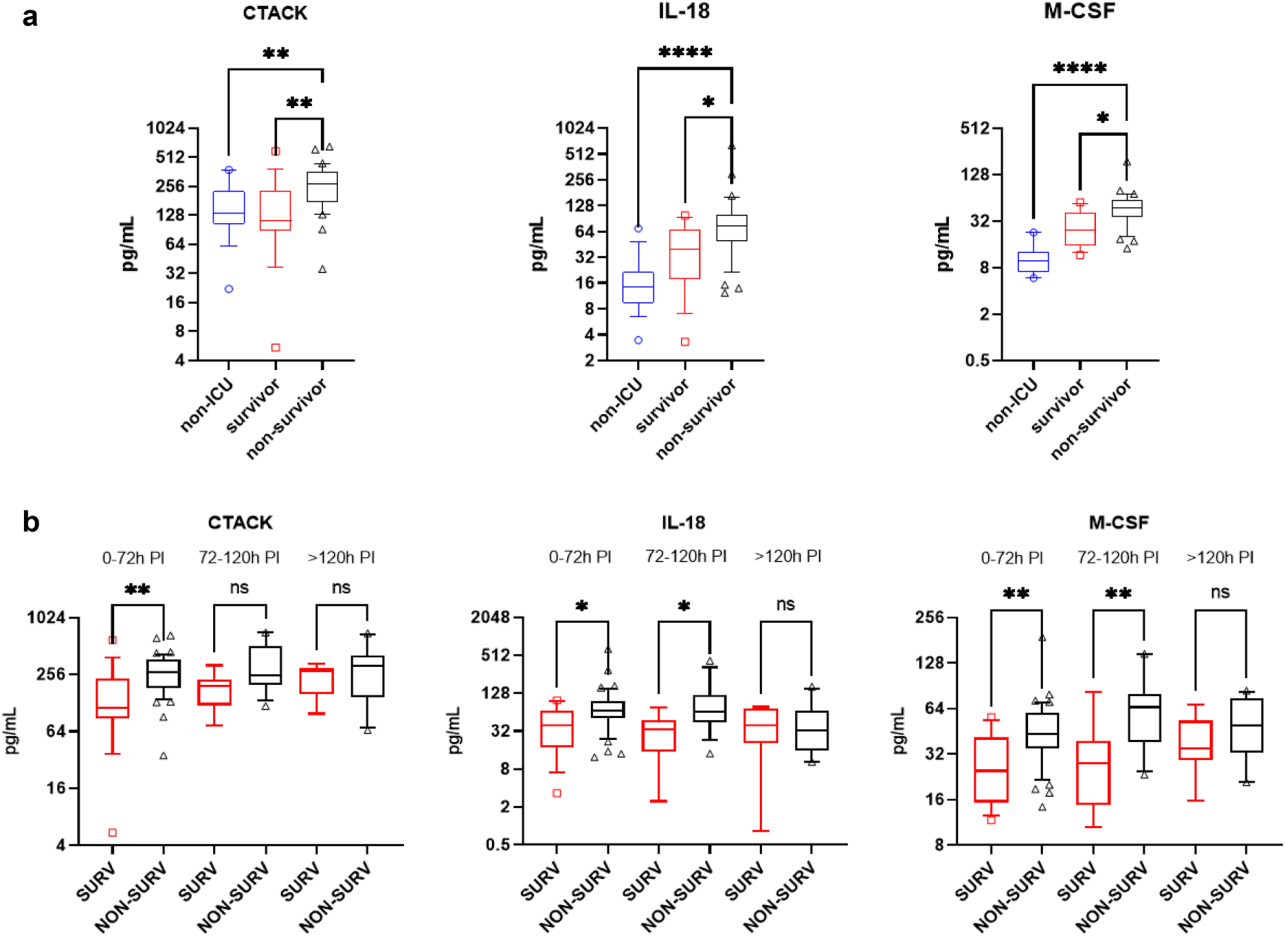
Analysis of mortality associated cytokines. (**a**) CTACK, IL-18 and M-CSF showed a significant increase in ICU non-survivors (black triangles) in comparison to ICU survivors (red squares). For analysis, a one-way ANOVA Kruskal–Wallis followed by Dunn’s correction was performed. Box plot showing the median of each group and the 10–90 percentile. (**b**) Individual samples of all patients were pooled according to their sampling period post-intubation (PI) and analyzed using the Kruskal–Wallis with Dunn’s correction test. The box plot shows the median of each group with the 10–90% percentiles. ns = non-significant; * = *p* < 0.05; ** = *p* < 0.01; **** = *p* < 0.0001.

By classifying our cohort according to the patient disease severity and outcome, increased M-CSF, CTACK and IL-18 levels in the non-survivor group were found in comparison to ICU survivors and non-ICU hospitalized patients (Fig. 3a). This finding was validated in the delta cohort (Fig. S4, a). Longitudinal measurements of these three cytokines during ICU stay showed in both cohorts that increased concentrations in non-survivors were found early after ICU admission (Fig. 3b, Fig. S4b). In conjunction, these findings indicate that elevated CTACK, M-CSF and IL-18 may have a role in staging and identifying SARS-CoV-2 disease outcomes at ICU admission despite different VoCs involved. Notably, analysis of samples of critically-ill non-COVID pneumonia patients with severe sepsis collected at ICU admission did not show a significant elevation of CTACK, M-CSF or IL-18 in non-survivors (Fig. S5), arguing that their elevated levels are an exclusive feature of COVID-19 infection.

### Critically-ill SARS-CoV-2 patients have contrasting pulmonary functional trajectories according to their survival, and correlates to IL-10 and TNF-beta serum concentration levels

**Figure 4:**
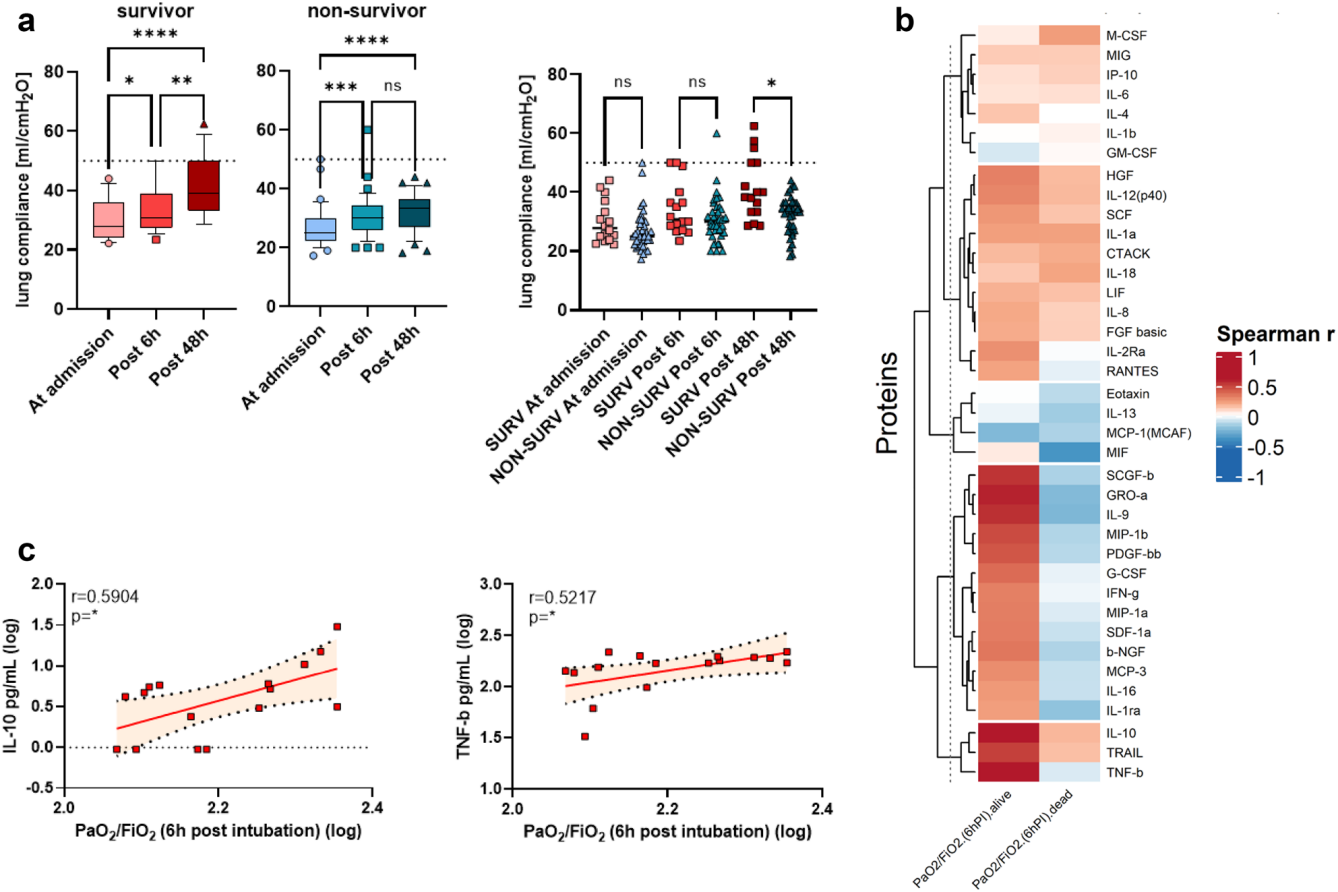
Analysis of cytokines and lung function. (**a**) Lung compliance was calculated for all patients. For analysis of survivors and non-survivors (left and middle graph) a Friedmann test or for the comparison of survivor to non-survivor (right graph) a one-way ANOVA Kurskal-Wallis followed by Dunn’s correction was performed. Box plots show the median of each group and the 10–90 percentile. Dot plots show the individual values of each patient (red = survivor, blue = non-survivor). The dotted line indicates 50 ml/cmH_2_O which is the lung compliance of a healthy individual. (**b**) Heatmap of Spearman correlation analysis of cytokines to the PaO_2_/FiO_2_ ratio at 6h post intubation. Red indicates positive correlation, blue negative correlation. (**c**) Individual correlation plot of PaO_2_/FiO_2_ ratio at 6h post intubation to IL-10 and TNF-β concentrations in ICU survivor. ns = non-significant; * = *p* < 0.05; ** = *p* < 0.01;*** = *p* < 0.001; **** = *p* < 0.0001.

The identification of IL-18 and M-CSF as severity-associated cytokines confirmed previous reports that correlate their increased systemic concentrations to worse outcomes^27,28^. However, CTACK had not been previously found to be associated with unfavorable clinical outcomes in severe COVID-19. As CTACK has recently been implicated in the pathogenesis of pulmonary fibrosis^29^, we decided to explore the pulmonary compartment in our cohort. By using non-invasive diagnostic imaging^30^, we found that around 80% of all pre-delta ICU patients presented fibro-proliferative changes by lung ultrasound sonography (USG) at ICU admission (Fig. S6). Moreover, by examining pulmonary functional data obtained from invasive respiratory monitoring, we found a significantly increased lung compliance in delta infected patients in comparison to pre-delta patients detected at ICU admission (Fig. S7b). When comparing the survivor and non-survivor groups in both cohorts, we identified contrasting functional trajectories depending on the VoC involved. In the pre-delta cohort, lung compliance was similar in survivor and non-survivor groups at admission, and survivors showed a significant improvement after 48 h of specialized intensive care (Fig. 4a). Conversely, delta infected patients showed hardly any improvement over time in both survivors and non-survivors (Fig. S7a) and had better lung compliance at admission in comparison to pre-alpha and alpha VoC (Fig. S7b). Remarkably, by examining the relationship between serum cytokine concentration and gas exchange at 6 h post intubation, we identified that IL-10 and TNF-beta serum levels had a significant positive correlation with PaO_2_/FiO_2_ in survivors of the pre-delta cohort (Fig. 4b,c). The delta cohort, however, showed much weaker correlation of cytokines to PaO_2_/FiO_2_ results (Fig. S7c). This evidence may suggest that patients that survived severe COVID-19 disease despite low initial lung compliance did not only show early differences in cytokine network regulation, but also in their functional pulmonary trajectories in comparison to those who did not survive. The contrasting findings seen in delta-infected patients may be a reflection of underlying differences in patients’ status at admission to the ICU, or of differences in the VoC afflicting them.

### Severe dysregulation of cytokine correlation in ICU non-survivor

**Figure 5:**
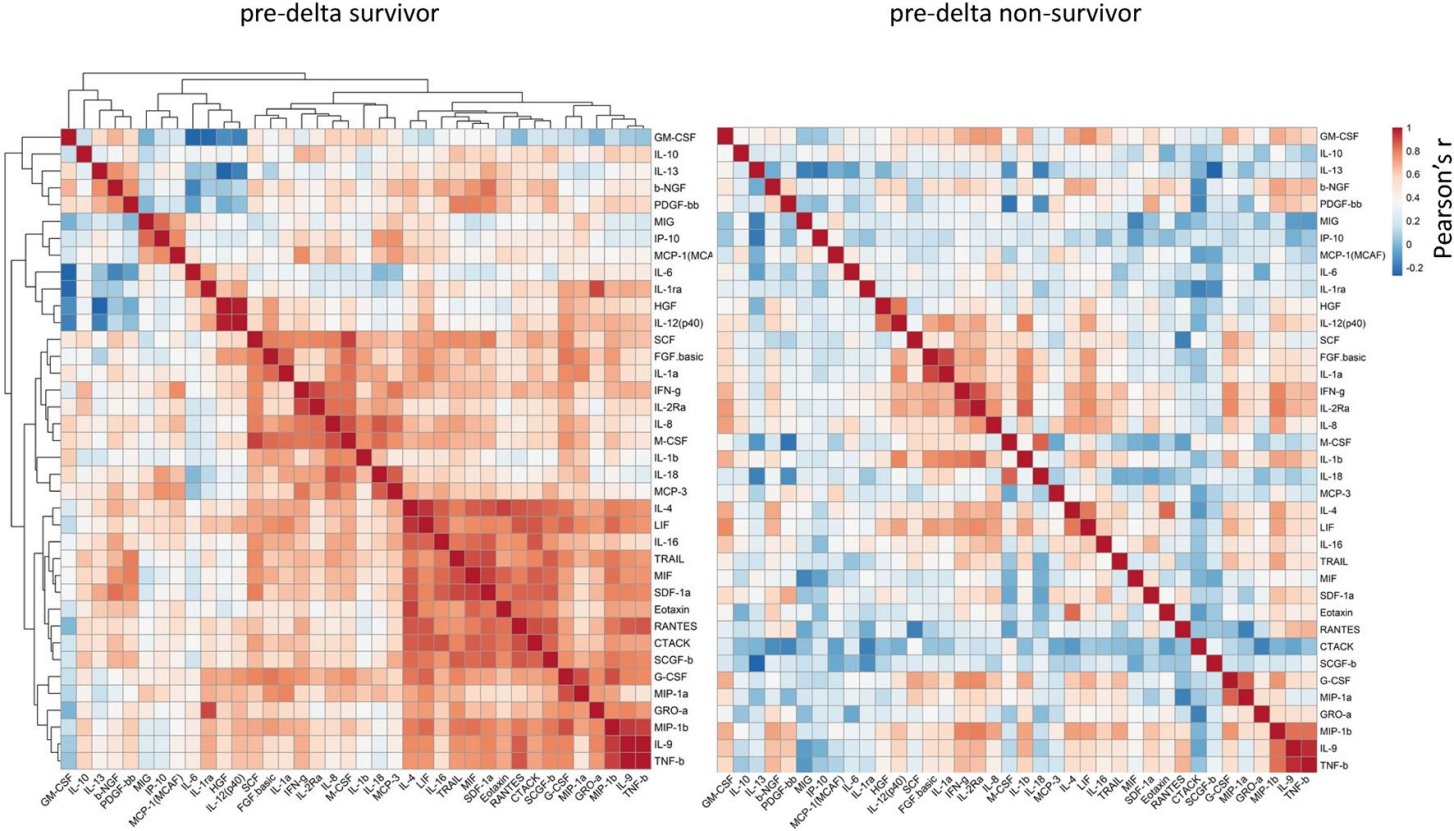
Cytokine-to-Cytokine correlation analysis. Cytokine concentrations were log transformed and a parametric Pearson correlation analysis was performed. Rows and columns represent cytokines. Red symbolized positive correlation and blue negative correlation. Cytokines were clustered according to Euclidean distance. The order of cytokines both in the rows and columns are equal between survivor and non-survivor. (**a**) Correlation heatmap of samples from the first time point (24-36h post intubation. (**b**) correlation heatmap of samples from the second time point (48-72h after the first sampling). Samples from 10/16 survivor and 20/38 non-survivor were available at the second time point due to earlier release from ICU or passing away from patients.

Cytokines regulate the inflammatory response via complex networks that balance their levels and ultimately their effects on the host^31^. To investigate this network in our cohort, we performed a cytokine-to-cytokine correlation analysis in the earliest samples from ICU admission. By comparing correlation heatmaps, a significant cytokine-to-cytokine association was found in survivors, which was almost entirely lost in the non-survivor group (Fig. 5). Importantly, this was also observed in the delta cohort, although to a lesser extent (Fig. S8). These results suggest that a severe dysregulation of the systemic cytokine network in COVID-19 non-survivors was already present at ICU admission. Importantly, the same cytokine correlation analysis in critically ill non-COVID patients with severe sepsis revealed an opposite pattern with an increase of correlation in non-surviving patients (Fig. S9).

## Discussion

Several serum cytokines have been associated with COVID-19 disease severity and have been proposed as potential biomarkers and/or therapeutic targets^32^. High IL-6, procalcitonin (PCT) and CRP were associated with fatal outcomes using samples collected at hospital admission in a German COVID-19 cohort with a mortality of around 11%^33^. By using a similar time point, another group found that IFN-β, IL-13, TNF-β, TGF-α, and IL-18 were associated with mortality^14^. Likewise, an international study in Europe identified serum HGF and CXCL13 as markers of mortality after confirmation by a validation cohort^8^. These differences in cytokine identification underline difficulties at comparing heterogeneous, low-mortality populations with inconstant hospitalization criteria across sites. For this reason, we decided to focus on a high-mortality cohort in which sampling occurred at ICU admission due to its temporal association with severe worsening of pulmonary and systemic compromise. This cohort underwent a strict standardized clinical management protocol that did not include immunomodulatory therapies, other than systemic steroid therapy, as recommended by international guidelines^34^. This led to a patient assortment that allowed a forthright survival classification with minimal treatment selection bias. Additionally, by using a second cohort, which was infected with another VoC, we were able to validate key findings while analyzing differences and similarities across variants. By dividing the ICU groups according to the fatal outcome after a 30-day follow-up, we found three cytokines that were significantly elevated in non-survivors (CTACK, IL-18 and M-CSF) at ICU admission, both in pre-delta and in delta infected patients but not in the non-COVID cohort. Besides association with mortality, we found a weak but significant correlation with serum SARS-CoV-2 viral load for CTACK in non-survivors of both cohorts, which may suggest a relationship between serum mediators and viral-induced pathogenicity, as an important survival determinant^18^. Remarkably, this cytokine level also positively correlated with APACHE II, a validated prediction tool used in ICU. Our study results are partly overlapping with those by Takashima et al.^35^, who investigated cytokine responses in a small cohort with a 43% mortality rate. They found that non-survivors had significantly elevated levels of several immune mediators including SDF-1, SCYB16, sCD30, IL-11, IL-18, IL-8, IFN-γ, TNF-α, sTNF-R2, M-CSF, and I-309. On the other hand, TRAIL levels were significantly lower in the cohort on mechanic ventilation than in the hospitalized controls, which was in line with previous reports ^36,37^, and possibly an indication of protective TRAIL effects.

The mortality-associated cytokines that we identified have various mechanistic and therapeutic significance. M-CSF is a growth factor implicated in steady-state mielopoiesis and monocyte production of proinflammatory cytokines^38^. A role of M-CSF in lung infection resolution has been reported, as animal models have confirmed its relevance in bacterial pneumonia and mononuclear phagocyte activation^39^. M-CSF has also been associated with lung damage in COVID-19^40,41^. IL-18 identification as a severity-associated cytokine brings further evidence of dysregulation in the monocyte/macrophage population. IL-18 is a member of the IL-1 family, mainly produced by macrophages and monocytes in response to several stimuli, including viral infection. By identifying IL-18 association with worse COVID-19 outcomes, our study is in line with previous reports^15,27,42^, and gives support to the role of monocyte/macrophage dysfunction and myeloid-driven immunopathology as COVID-19 severity determinants^9^. Interestingly, anti-IL-18 antibodies have been tested in clinical settings and shown to reduce inflammatory markers like CRP but not APACHE II scores, when compared with placebo treated patients^43^. This shows the relevance of this cytokine but also indicates that IL-18 alone is not the key driver of decease severity. Importantly, by using a cohort of non-COVID-19 patients with sepsis and pneumonia as control, we delineated the specificity of these analytes for severe SARS-CoV-2 disease.

We identified a hitherto undescribed association of systemic CTACK levels with poor prognosis of COVID-19. CTACK has been thought to be exclusively involved in skin-related responses until recently. Its role as a biomarker in idiopathic lung fibrosis (ILF), a rapidly progressive interstitial pneumonia associated with alveolar epithelial injury and abnormal tissue repair without a known cause, has been identified^29^. One small COVID-19-related study reported an increase of CTACK in COVID-19 patients in comparison to healthy controls but concentrations did not differ among severity degrees^44^. While fibrotic pulmonary complications have been described as COVID-19 sequelae^44,45^, no investigation on its relation to acute severe COVID-19 has been performed. In the pre-delta cohort, the majority of ICU patients presented a low lung compliance at ICU admission, as well as a fibroproliferative pattern identified by lung ultrasound. Interestingly, patients infected with the later occurred VoC delta did not show this low lung compliance. These findings may constitute evidence of early fibrosis-like changes within lungs of severely ill patients infected with pre-delta VoCs. Of interest, a transcriptomic tool formulated to predict ILF outcomes was also useful for COVID-19^46^, suggesting a shared pathophysiology. While an association of cytokine networks to lung fibrosis remains a topic for future investigations, our findings constitute to our knowledge the first report on CTACK association to fatal COVID-19 outcomes.

Finally, we found differences in cytokine-to-cytokine correlation between survivors and non-survivors. Cytokine regulation involves an enhancer-suppressor network that has been proposed to be defective in severe COVID-19, as uncontrolled cytokine overproduction is a key driver of COVID-19 severity^47^. Our work shows that the loss of cytokine-to-cytokine correlation is present at ICU admission in patients who did not survive, supporting the hypothesis that an early immune dysregulation profile may have a prognostic value or be a therapeutic target, besides the administration of systemic steroids. This finding is supported by a study which also proposed a dysregulated cytokine response at ICU admission to be associated with mortality^48^. It is remarkable that this disruption of cytokine networks was specific for our COVID-non-survivors and not a feature encountered in non-COVID pneumonia casualties. While our dataset do not allow a cause-effect analysis, it identified novel cytokines as indicators of COVID-19 outcomes.

## Conclusion

To conclude our findings, even though pre-delta and delta patients presented differences in clinical and laboratory markers, the cytokine profiles showed similar patterns. The novel mortality-associated cytokine CTACK showed in both cohorts associations to mortality and severity-based markers. Additionally, IL-18 and M-CSF have been found across other studies to be severity/ mortality associated, nevertheless, a clear answer is still not found which cytokines are actually responsible for mortality. Besides those individual cytokines, we report an early loss of cytokine-to-cytokine correlation in the non-survivor groups. These findings might point to the importance of cytokine detection even for Omicron strains of SARS-CoV-2 that have prevailed recently, which would need to be confirmed in independent cohorts.

### Limitations

Our study has several limitations. Sampling occurred in late 2020 and early and late 2021, prior to the onset of the omicron variants and prior to the emergence of a basal herd immunity. Hence, it is possible that the immune responses may skew differently in hosts that are primed against SARS-CoV-2 and/or infected with more recent VoC. Since our data were collected on a cohort of mechanically ventilated patients, where PaO_2_/FiO_2_ ratios may be affected by FiO_2_ applied to patients^49^, the correlation of cytokine to PaO_2_/FiO_2_ levels needs to be considered carefully. Similar to other discovery-based cohorts, our sample size was not adequately powered, and it was dependent on patient availability and logistical limitations. In addition, although multiplex assay allows for a broad immune mediator screening, confirmation of our mortality-related cytokines significance in other platforms is desirable in subsequent validation cohorts. Nevertheless, our results provide a direct insight into the systemic cytokine networks at a critical time point in COVID-19 and identify CTACK as a novel mortality-associated cytokine in pre-delta and delta infected patients, which may have important mechanistic and therapeutic implications.

### Supplementary Information

Supplementary Information 1.

Supplementary Information 2.

Supplementary Information 3.

Supplementary Information 4.

Supplementary Information 5.

## Data Availability

The datasets used and/or analyzed during the current study are available from the corresponding author on reasonable request**.**
