# Supplementary material for: Serum cytokine dysregulation signatures associated with COVID-19 outcomes in high mortality intensive care unit cohorts across pandemic waves and variants
Source: Sci Rep. 2024 Jun 13;14:13605. doi: 10.1038/s41598-024-64384-y (PMC11176334; doi:10.1038/s41598-024-64384-y)
Supplement: Supplementary file 1 — Supplementary Information 1. [file 41598_2024_64384_MOESM1_ESM.docx]

# **Supplementary figures to**

# **Serum cytokine dysregulation signatures associated with COVID-19 outcomes in high mortality intensive care unit cohorts across pandemic waves and variants**

# **Authors**

Henrike Maaß^1,2*^, Mario Ynga-Durand^1,2*^, Marko Milošević^3^, Fran Krstanović^4^, Marina Pribanić Matešić^4^, Iva Žuža^5^, Stipan Jonjić^4^, Ilija Brizić^4^, Alan Šustić^3,6^, Frank Bloos^7^, SepNet Critical Care Trials Group^8^, Alen Protić^3^, Luka Čičin-Šain^1,2,9**^

^1^ Department of Viral Immunology, Helmholtz Center for Infection Research, Braunschweig, Germany

^2^ Centre for Individualized Infection Medicine (CiiM), a joint venture of Helmholtz Centre for Infection Research and Hannover Medical School, Hannover, German

^3^ Department of Anesthesiology, Reanimation, Intensive Care and Emergency Medicine, Faculty of Medicine, University of Rijeka, Rijeka, Croatia

^4^ Center for Proteomics, Faculty of Medicine, University of Rijeka. Rijeka, Croatia

^5^ Department of Radiology, Clinical Hospital Centre Rijeka, Croatia

^6^ Department of Clinical Medical Science II, Faculty of Health Studies, University of Rijeka. Rijeka, Croatia

^7^ Jena University Hospital, Dept. of Anesthesiology and Intensive Care Medicine, Jena, Germany

^8^ The full list of SepNet Critical Care Trials Group (SepNet) authors are shown in the Additional file 2

^7^ German Centre for Infection Research (DZIF), partner site Hannover/Braunschweig, Braunschweig, Germany

^*^ These authors contributed equally

^**^ Correspondence: Luka.Cicin-Sain@helmholtz-hzi.de

# **Contents**

**Table S1**: Demographic table of delta cohort

**Table S2**: Demographic table of non-COVID cohort

**Figure S1:** Viral load comparison of patients infected with different VoC.

**Figure S2**: Cytokine analysis of delta COVID-19 patients

**Figure S3**: Cytokine association to clinical marker in the delta cohort

**Figure S4**: Mortality associated cytokines of the delta cohort.

**Figure S5**: Identified SARS-CoV-2 mortality associated cytokines in non-COVID sepsis patients

**Figure S6**: Detection of fibroproloferative changes in the lung of pre-delta COVID-19 patients

**Figure S7**: Lung function analysis of the delta cohort

**Figure S8**: Cytokine-to-Cytokine correlation analysis of validation cohort

**Figure S9**: Cytokine-to-cytokine correlation analysis of non-COVID cohort

**Table S1**: Demographic table of delta cohort

|  | **ICU Non-Survival** | **ICU Survival** | ***p* between Non-Survival and Survival Groups)** |
| --- | --- | --- | --- |
|  | **(*n* = *24*)** | **(*n* = *9*)** |  |
| **Demographic characteristics** |  |  |  |
| Female % | 37.5 | 22.2 | ns **^a^** |
| Age in years (Mean ± SD) | 68 ± 8.04 | 55 ± 14.6 | 0.002 **^b^** |
| Sample per patient (Mean ± SD) | 1.79 ± 0.93 | 1.89 ± 0.78 | ns **^c^** |
| **Clinical characteristics** |  |  |  |
| PaO_2_/FiO_2_ at ICU admission (Mean ± SD) | 126.2 ± 42.7 | 160 ± 68.2 | ns **^b^** |
| Moderate-Severe ARDS at admission (%) | 92 | 89 | ns **^a^** |
| SOFA score at admission (Mean ± SD) | 7.2 ± 2 | 3.8 ± 1.2 | < 0.0001 **^b^** |
| APACHE II score at admission (Median, p25–p75) | 18.5 (14-22) | 6 (4.5-17) | 0.0009 **^c^** |
| Fever at admission (%) | 22.22 | 25 | ns **^a^** |
| ICU stay length in days (Median, p25–p75) | 6 (4-9) | 12 (8.5-21) | 0.0055 **^c^** |
| SARS-CoV-2 Immunization started (%) | 8.3 | 11.1 | ns **^a^** |
| **Comorbidities** |  |  |  |
| Coronary heart disease (%) | 12.5 | 11.11 | ns **^a^** |
| Hypertension (%) | 62.5 | 33.33 | ns **^a^** |
| Diabetes (%) | 25 | 0 | ns **^a^** |
| Obesity (%) | 41.67 | 22.22 | ns **^a^** |
| Cancer (%) | 12.5 | 11.11 | ns **^a^** |
| Chronic respiratory disease (%) | 20.83 | 11.11 | ns **^a^** |
| Immunosuppression (%) | 0 | 0 | ns **^a^** |
| Number of comorbidities (Median, p25-p75) | 2 (1-2) | 1 (0.5-1.5) | 0.0329 **^c^** |
| **Laboratory Markers** |  |  |  |
| **at Admission** |  |  |  |
| WBC c/uL x10^6^ (Mean ± SD) | 11.05 ± 5.72 | 10.48 ± 4.7 | ns **^b^** |
| Hemoglobin g/L (Mean ± SD) | 139.2 ± 19.64 | 137.7 ± 14.47 | ns **^b^** |
| CRP mg/dL (Mean ± SD) | 178.1 ± 102.4 | 181.5 ± 105.7 | ns **^b^** |
| **Treatment during ICU** |  |  |  |
| **hospital stay** |  |  |  |
| Days on mechanical ventilation (Median, p25–p75) | 6 (4-9) | 8 (4-17) | ns **^c^** |
| Shock (%) | 66.7 | 11.1 | 0.0066 **^a^** |
| Days on vasopressors and/or inotropics (Median, p25–p75) | 2.5 (1-4) | 0 (0-5.5) | ns **^c^** |
| Renal Replacement therapy use (%) | 4.17 | 11.11 | ns **^a^** |
| Ventilator-associated pneumonia (%) | 41.67 | 16.67 | ns **^a^** |
| Catheter-associated bloodstream infection (%) | 0 | 11.11 | ns **^a^** |

ns = non-significant, ^a^ Fisher’s exact test, ^b^ unpaired t test, ^c^ Mann-Whitney test

**Table S2**: Demographic table of non-COVID cohort

|  | **ICU Non-Survival** | **ICU Survival** | ***p* between Non-Survival and Survival Groups)** |
| --- | --- | --- | --- |
|  | **(*n* = *5*)** | **(*n* = *15*)** |  |
| **Demographic characteristics** |  |  |  |
| Female % | 0 | 20 | ns ^a^ |
| Age in years (Mean ± SD) | 71 ± 2.35 | 69.4 ± 2.35 | ns ^b^ |
| **Clinical characteristics** |  |  |  |
| PaO_2_/FiO_2_ at ICU admission (Mean ± SD) | 151.6 ± 79.48 | 127.4 ± 60.96 | ns ^b^ |
| Moderate-Severe ARDS at admission (%) | 40 | 13.33 | ns ^a^ |
| SOFA score at admission (Mean ± SD) | 10.3 ± 2.98 | 11 ± 1.51 | ns ^b^ |
| APACHE II score at admission (Median, p25–p75) | 27.73 ± 6.28 | 29.86 ± 7.56 | ns |
| Fever during ICU stay (%) | 30 | 70 | ns ^a^ |
| ICU stay length in days (Median, p25–p75) | 19.18, 12-24 | 24.78, 15-34 | ns ^c^ |
| SARS-CoV-2 Immunization started (%) | 0 | 0 | ns ^a^ |
| **Comorbidities** |  |  |  |
| Coronary heart disease (%) | 36.36 | 22.22 | ns ^a^ |
| Hypertension (%) | no data |  |  |
| Diabetes (%) | 36.36 | 11.11 | ns ^a^ |
| Obesity (%) | 9.09 | 11.11 | ns ^a^ |
| Cancer (%) | 45.45 | 44.44 | ns ^a^ |
| Chronic respiratory disease (%) | 18.18 | 22.22 | ns ^a^ |
| Immunosuppression (%) | 0 | 0 | ns ^a^ |
| Number of comorbidities (Median, p25-p75) | no data |  |  |
| **Laboratory Markers** |  |  |  |
|  |  |  |  |
| Max WBC during ICU stay c/uL x10^6^ (Mean ± SD) | 20.84 ± 12.83 | 14.8 ± 7.79 | ns ^b^ |
| Min Hemoglobin during ICU stay, g/dL (Mean ± SD) | 9.87 ± 2 | 11.94 ± 2.77 | ns ^b^ |
| Max CRP during ICU stay, mg/dL (Mean ± SD) | 163.3 ± 154.8 | 129.4 ± 116.2 | ns ^b^ |
| **Treatment during ICU** |  |  |  |
| **hospital stay** |  |  |  |
| Days on mechanical ventilation (Median, p25–p75) | 16, 10-22 | 18, 12-28 | ns ^c^ |
| Shock (%) | 60 | 100 | ns ^a^ |
| Days on vasopressors and/or inotropics (Median, p25–p75) | 11, 5-19 | 6, 4-15.5 | ns ^c^ |
| Renal Replacement therapy use (%) | 36.36 | 11.11 | ns ^a^ |
| Ventilator-associated pneumonia (%) | no data |  |  |
| Catheter-associated bloodstream infection (%) | 9.09 | 11.11 | ns ^a^ |

ns = non-significant, ^a^ Fisher’s exact test, ^b^ unpaired t test, ^c^ Mann-Whitney test

**Fig. S1: Viral load comparison of patients infected with different VoC.** a) Serum and BAL samples of SARS-CoV-2 delta-VoC infected patients on ICU were analysed in regards of viral load. Patients were divided by their mortality outcome (SURV=survival, NON-SURV=non-survival). Student t test was performed on samples with the highest viral load per patient and earliest sample available per patient. b) SARS-CoV-2 variants were identified and viral load in serum and BAL was compared according to different VoC. Violin plots of median and quartile SARS-CoV-2 copy numbers of individuals are shown. Linear regression with adjustment for age was performed on samples with the highest viral load per patient and earliest sample available per patient. Blue circles show data for pre-alpha infected patients, red squares for alpha and black triangles for delta infected patients. Asterisks indicate significant differences between groups. ns=non-significant; ns=non-significant; *=p<0.05; **=p<0.01.

**Fig. S2**: **Cytokine analysis of delta COVID-19 patients.** Heatmap of log-transformed cytokine concentrations of serum samples of the earliest time points. Patients are represented per column and patient conditions are indicated by column annotations. Cytokines are represented by rows. Clustering was performed based on Euclidean distance. ns=non-significant; *=p<0.05.

**Fig. S3:** **Cytokine association to clinical marker in the delta cohort.** a) Spearman correlation analysis of cytokines to hemoglobin (HB) values, white blood cell count (WBC) and C reactive protein (CRP) was performed. Red indicates positive correlation, blue negative correlation. b) Waterfall plot of Spearman correlation from cytokines to APACHE II scores. The dotted line indicates values above r_S_=0.35 and cytokines with statistically significant correlation (IL-16 (blue), IL-1a (orange), IL-1ra (magenta) and CTACK (green)). c) Correlation of CTACK to SARS-CoV-2 viral load in serum samples of ICU non-survivors (black). Pearson analysis was performed. Linear-regression is shown as well as the 90% CI indicated by the dotted lines. ns=non-significant; *=p<0.05.

**Fig. S4: Mortality associated cytokines of the delta cohort**. a) CTACK, IL-18 and M-CSF showed a significant increase in ICU non-survivors (black triangles) in comparison to ICU survivors (red squares). b) Individual samples of all patients were pooled according to their sampling period post intubation (PI). Due to a low amount of samples from time points > 72h PI and the loss of significant power, no analysis of these time points were performed. Depending on the outcome of the Shapiro test, a Mann-Whitney test (IL-18, M-CSF) or Welch’s test (CTACK) was performed. The box plot shows the median of each group with the 10-90% percentiles. ns=non-significant; *=p<0.05; **=p<0.01;***=p<0.001.

**Fig. S5: Identified SARS-CoV-2 mortality associated cytokines in non-COVID sepsis patients.** CTACK, IL-18 and M-CSF showed no significant increase in non-COVID non-survivors (triangles) in comparison to non-COVID survivors (squares). Welch’s test was performed. The box plot shows the median of each group with the all data points. ns=non-significant.

**Fig. S6: Detection of fibroproloferative changes in the lung of pre-delta COVID-19 patients**. Lung ultra-sonography was performed on 11/16 survivor and 32/38 non-survivor patients. Images were scored according to [1]. Box plot showing the median of each group and the whiskers minimum and maximum values. Dotted line indicated a LUSCORE of 7, lungs with values >7 were identified as fibroproliferative changes.

**Fig. S7: Lung function analysis of the delta cohort.** a) Lung compliance was calculated for all patients. For analysis of survivor and non-survivor a Friedmann test or for the comparison of survivor to non-survivor a one-way ANOVA Kurskal-Wallis followed by Dunn’s correction was performed. Box plot showing the median of each group and the 10-90 percentile. Dot plot shows the individual values of each patient (red=survivor, blue=non-survivor). The dotted line indicates 50 ml/cmH_2_O which is the lung compliance of an health individual. b) Comparison of lung compliance at different time points post intubation of patients infected with VoC pre-alpha (blue), alpha (red) and delta (black). Violin plots of median and quartile are shown. Linear regression with age adjustment was performed. c) heatmap of Spearman correlation analysis of cytokines to the PaO_2_/FiO_2_ ratio at 6h post intubation. Red indicates positive correlation, blue negative correlation. ns=non-significant; *=p<0.05; **=p<0.01;***=p<0.001.

**Fig. S8:** **Cytokine-to-Cytokine correlation analysis of validation cohort.** Cytokine concentrations of delta infected patients were log transformed and a parametric Pearson correlation analysis was performed. Rows and columns represent cytokines. Red symbolized positive correlation and blue negative correlation. Cytokines were clustered according to Euclidean distance. The order of cytokines both in the rows and columns are equal between survivor and non-survivor. Correlation heatmap of samples from the first time point (24-36h post intubation).

**Fig. S9: Cytokine-to-cytokine correlation analysis of non-COVID cohort**. Cytokine concentrations of non-COVID patients were log transformed and a parametric Pearson correlation analysis was performed. Rows and columns represent cytokines. Red symbolized positive correlation and blue negative correlation. Cytokines were clustered according to Euclidean distance. The order of cytokines both in the rows and columns are equal between survivor and non-survivor.

# **References**

1. de Almeida Monteiro RA, Duarte-Neto AN, Ferraz da Silva LF, de Oliveira EP, do Nascimento ECT, Mauad T, Saldiva P, Dolhnikoff M, (2021) Ultrasound assessment of pulmonary fibroproliferative changes in severe COVID-19: a quantitative correlation study with histopathological findings. Intensive Care Med 47: 199-207
