# Supplementary material for: Serum cytokine dysregulation signatures associated with COVID-19 outcomes in high mortality intensive care unit cohorts across pandemic waves and variants
Source: Sci Rep. 2024 Jun 13;14:13605. doi: 10.1038/s41598-024-64384-y (PMC11176334; doi:10.1038/s41598-024-64384-y)
Supplement: Supplementary file 2 — Supplementary Information 2. [file 41598_2024_64384_MOESM2_ESM.docx]

# SepNet Critical Care Trials Group

(pubmed listed, and ordered alphabetically by location)

| Affiliation | Name | Location |
| --- | --- | --- |
| University Hospital RWTH Aachen, Department of Surgical Intensive Care Medicine and Intermediate Care | Gernot Marx, MD | Aachen |
| University Hospital Augsburg, Dept. of Anesthesiology and Surgical Intensive Care Medicine | Ulrich Jaschinski, MD | Augsburg |
| Charité Berlin, Department of Anesthesiology, Division of Operative Intensive Care Medicine | Konrad Reinhart, MD; Claudia Spies, MD | Berlin |
| Vivantes Hospital Neukölln, Department of Cardiology and Intensive Care Medicine | Lorenz Reil, MD | Berlin |
| University Hospital Bonn Division of Intensive Care Medicine, Dept. of Anesthesiology and Intensive Care Medicine | Christian Putensen, MD | Bonn |
| University Hospital Carl Gustav Carus, Dept. of Anesthesiology and Intensive Care Medicine | Maximilian Ragaller, MD | Dresden |
| University Hospital Freiburg, Department of General Surgery | Stefan Utzlino, MD | Freiburg |
| Dept. of Anesthesiology, University Medical Center, Georg-August-University Göttingen^1^ | Onnen Mörer, MD | Göttingen |
| University Hospital Greifswald, Dept. of Anesthesiology and Intensive Care Medicine^1^ | Matthias Gründling, MD | Greifswald |
| University Hospital Hamburg-Eppendorf, Dept. of Intensive Care Medicine^1^ | Stefan Kluge, MD, Axel Nierhaus, MD | Hamburg |
| University Hospital Hannover, Dept. of Pneumology and Infectiology | Tobias Welte, MD | Hannover |
| Jena University Hospital, Dept. of Anesthesiology and Intensive Care Medicine | Michael Bauer, MD; Frank Bloos, MD; Katrin Ludwig, MD | Jena |
| Jena University Hospital, Institute of Clinical Chemistry and Laboratory Diagnostics | Michael Kiehntopf, MD | Jena |
| University Hospital Schleswig-Holstein – Campus Kiel, Department of Anesthesiology and Intensive Care Medicine | Gunnar Elke, MD | Kiel |
| University Lepizig, Center for Clinical Studies | Holger Bogatsch; Christoph Engel; Markus Loeffler | Leipzig |
| University Hospital Munich, Department of Anesthesiology | Josef Briegel, MD; Ines Kaufmann | Munich |
| Hospital Nuremberg, Department of Internal Medicine, Division Cardiology | Stefan John, MD | Nuremberg |
| University Hospital Tübingen, Department of Internal Medicine | Reimer Riessen, MD | Tübingen |
| University Hospital Würzburg, Department of Anaesthesiology, Intensive Care, Emergency and Pain Medicine | Patrick Meybohm | Würzburg |
