# Supplementary material for: Serum cytokine dysregulation signatures associated with COVID-19 outcomes in high mortality intensive care unit cohorts across pandemic waves and variants
Source: Sci Rep. 2024 Jun 13;14:13605. doi: 10.1038/s41598-024-64384-y (PMC11176334; doi:10.1038/s41598-024-64384-y)
Supplement: Supplementary file 5 — Supplementary Information 5. [file 41598_2024_64384_MOESM5_ESM.docx]

**List of Abbrevations**

| **APACHE II score** | **Acute Physiology and chronic health evaluation** |
| --- | --- |
| **ARDS** | **Acute respiratory distress syndrome** |
| **BAL** | **Bronchoalveolar lavage** |
| **COVID-19** | **Coronavirus diseasem 2019** |
| **CRP** | **C reactive protein** |
| **ET** | **Endotracheal tube** |
| **HB** | **Hemoglobin** |
| **ICU** | **Intensive care unit** |
| **ILF** | **Idiopathic lung fibrosis** |
| **IMV** | **Invasive mechanical ventilation** |
| PaO_2_/FiO_2_ | **Horowitz index** |
| **PI** | **Post-intubation** |
| **RT-qPCR** | **Reverse-transriptase quantitative PCR** |
| **SARS-CoV-2** | **Severe acute respiratory syndrome virus 2** |
| **SD** | **Standard deviation** |
| **SOFA score** | **Sepsis-related organ failure assessment score** |
| **USG** | **Ultrasound sonography** |
| **VoC** | **Variant of concern** |
| **WBC** | **White blood cell count** |
